# Concept of molecular prevention

**DOI:** 10.1186/1878-5085-5-S1-A48

**Published:** 2014-02-11

**Authors:** Christoph A Karle, Giang Duong, Thomas M Helms

**Affiliations:** 1 Medical office for diagnosis, Kuenzelsau, Germany; 2 German Foundation for the Chronically Ill, Fuerth , Germany

## 

Prior to the development of a disease, Preventive Medicine enables early detection of signs and symptoms of diseases; if logical measures are taken, an outbreak i.e the development of a disease may possibly be avoided. If the possibility of curing a disease is missed, only the treatment and relief of symptoms remains. The disease becomes incurable and chronic. Since the healing of serious illnesses is only effective as long as the patient is not yet seriously affected, the concept of healing should be redefined. Thus, the removal of a small tumor, the patient hasn't yet noticed, could be considered to fall into the category of preventive medicine (see Figure 1). The basic principle can be illustrated particularly well by the example of a tumor, but applies to all diseases. It follows, that prevention, i.e. the early detection of disease, is the most effective method to combat disease. Since many predisposing factors for disease development are already defined during embryonic development, a lot of time is left to carry out successful prevention.

**Figure 1:**
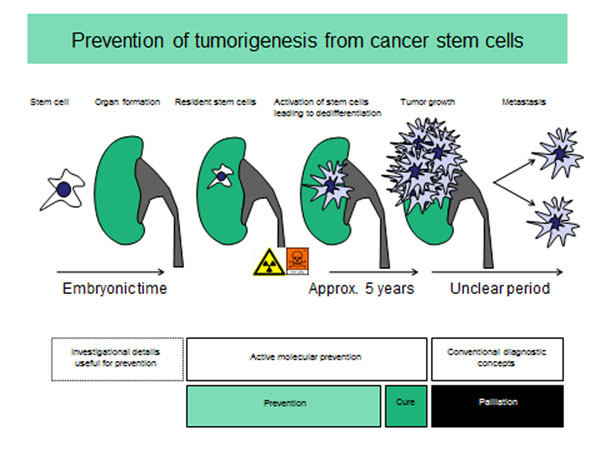
Concept - pathogenesis of renal cell cancer. Vulnerable residual stem cells, which, after being inflicted by damaging effects, do not have appropriate repair mechanisms and are therefore transformed into so-called tumor stem cells. In the presented model, the vulnerability of the cells is already genetically determined and thus theoretically predictable. After initiation of tumor growth, healing can only be achieved if no significant metastasis has occurred and later through the removal of the tumor. Partial control of metastasis by the body's immune system is suspected.

Modern preventive medicine makes use of all technical means of modern diagnostics - from the extensive history up to detailed genetic investigations. It examines all relevant organ systems according to the WHO (World Health Organization) defined hierarchical table of the most common life-threatening causes of diseases. Physical exams - with the help of equipment - allow an assessment of a patient's condition at the time of the investigation.

Sustainability, that is the period for which the test results are valid, can be estimated from the results of such studies and from the observation period of the study. Since there is no scientific advantages or post-closure plan for many diseases, routine follow-up examination are usually scheduled at set, agreed upon intervals, e.g. once a year.

If a more accurate assessment of the disease risk in healthy patients is desired or the prognosis of a particular course of a known disease with known genetic factors favoring the disease is suspected, genetically predisposing factors should be checked. This is done by means of molecular genetic testing of possibly afflicted genes in the DNA of the patient. After creating an exact genetic fingerprint, worldwide comparisons of the genetic fingerprints among patients with the same or similar changes can be performed and conclusions can be drawn and predictions can be made.

High quality laboratory equipment allows both targeted hotspot analysis (using the Sanger sequencing) as well as screening tests on several hundred genes (using the panel study of next-generation sequencing).

For the screening tests, validated predisposing-genes are examined for the following disease groups:

a.) Heart and vascular diseases

b.) Tumors

c.) Metabolic diseases

d.) Diseases of bones and connective tissues

e.) Diseases of the sense organs

f.) Neurodegenerative Diseases

g.) Organ and systemic diseases

h.) Blood diseases

The use of molecular genetic studies is subject to strict guidelines that are laid down in the so-called Gene-Diagnosis-Law. For this reason, human genetic consultations are prescribed by a specialist in human genetics before performing predictive diagnostics on healthy patients. Human genetic counseling before proceeding with the diagnostic methods may be omitted if there is the suspicion of a genetically determined disease upon physical or other examinations.

The overall concept includes subtle diagnostic methods in molecular medicine, particularly translational research techniques which have not yet become standard in the medical routine examinations. Therefore, we have coined the term "molecular prevention" for it.

## Investigational methods

Blood tests (blood count, blood chemistry, lipids, CRP, PSA)

Urinalysis

Physical Examination

(Blood pressure measurement)

(Skin inspection)

Exercise stress test, sports medicine and performance

Pulmonary function tests

Resting ECG

Exercise ECG

4-D echocardiography

Carotid duplex

Thyroid ultrasound

Upper abdominal ultrasound

Examination of the leg veins

(Plethysmography)

(Venous plethysmography)

(Strain gauge plethysmography)

Examination of the leg arteries

(Pulse oscillography)

Bone density measurements

Eye examinations vision control

Ocular pressure

Photograph of the fundus

Ocular coherence tomography

## Optional

Capsule endoscopy of the entire gastrointestinal tract

Sleep apnea screening

Holter-monitor

Long-term blood pressure

Long-term glucose measurement

Cardiac MRI as a non-invasive coronary-diagnostic measure

Genetic testing for disease genes

Duration of basic checks: about 3.5 hours.

## Personalized conclusions from the findings

For each patient, a wealth of research data is generated that allows the development of a personalized strategy. However, the analysis of data allows not only the determination of a precise medical treatment strategy, but also the precise definition of reasonable care intervals, the observance makes a continuous monitoring of disease development / progress possible. In addition, the data serves as a basis for life style consultation to avoid specific hazards for which the body of the individual patient holds inadequate compensation mechanisms. Personalized exercise and nutrition concepts help patients to remain healthy.

